# Conducting Polyaniline Nanowire and Its Applications in Chemiresistive Sensing

**DOI:** 10.3390/nano3030498

**Published:** 2013-08-07

**Authors:** Edward Song, Jin-Woo Choi

**Affiliations:** 1 School of Electrical Engineering and Computer Science, Louisiana State University, Baton Rouge, LA 70803, USA; E-Mail: esong1@tigers.lsu.edu; 2 Center for Advanced Microstructures and Devices, Louisiana State University, Baton Rouge, LA 70803, USA

**Keywords:** polyaniline, nanowire, conducting polymer, chemiresistive

## Abstract

One dimensional polyaniline nanowire is an electrically conducting polymer that can be used as an active layer for sensors whose conductivity change can be used to detect chemical or biological species. In this review, the basic properties of polyaniline nanowires including chemical structures, redox chemistry, and method of synthesis are discussed. A comprehensive literature survey on chemiresistive/conductometric sensors based on polyaniline nanowires is presented and recent developments in polyaniline nanowire-based sensors are summarized. Finally, the current limitations and the future prospect of polyaniline nanowires are discussed.

## 1. Introduction

The accurate detection and quantification of chemical and biological species are of great importance in many areas including health care, environmental monitoring, and recently, defense against biological warfare. The key features of an effective sensor are that, in addition to having high sensitivity and selectivity, the detection method must be simple, rapid, and the sensor must be multiplexed for parallel detection of multiple target analytes [1]. However, existing technologies have not been able to demonstrate such sensing capability.

Nanomaterial-based sensors seem to be promising in this regard and have received great attention in recent years due to many unique properties that nanoscale materials offer. The main advantages of nanoscale materials include high surface-to-volume ratio leading to fast reaction speed and sensitivity, ease of miniaturization, minimum power consumption, and low cost as a result of the small volume of required reagents.

Chemiresistive or conductometric sensors have attracted a great deal of attention because of their simple structure and their ability to be miniaturized to give high density and high throughput sensor arrays. Chemiresistive or conductometric sensors measure the change in the conductance or resistance of the sensing material caused by its interaction with the target analytes. Chemiresistive sensors have the benefit of being simple in configuration as well as in signal measurement.

One dimensional nanomaterial-based chemiresistors can achieve high sensitivity and fast response while preserving simplicity and high density. In particular, nanotubes, nanowires, or nanorods are ideal for this configuration [2]. First of all, the small cross-sectional area of the nanowires maximizes the current response along the axial direction of the wires creating a large conductance change. Secondly, the large surface area of the nanowires improves the sensitivity of the nanowire-based sensor by increasing the chance of target analytes reacting with the surface of the nanowire. Thirdly, the direct conversion of the chemical change into an electrical signal greatly simplifies the device configuration. Finally, the nanoscale of the sensing material enables the development of high density, high throughput, and individually addressable sensor arrays for simultaneous multi-analyte detection. Reported results show that the large surface area of 1-dimensional nanowire network-based sensors improved the sensitivity compared to the conventional 2-dimensional films [3,4]. Recently, label-free detection of analytes using chemiresistive nanowire-based sensors has gained much interest due to its high sensitivity and real-time monitoring with a fast transduction mechanism [1,5,6]. The use of nanowires has been applied in biosensors as well [7].

Because of these advantages that the 1-dimensional nanowires provide in sensing capabilities, many types of nanowires have been studied as a sensing material such as carbon nanotubes [8,9,10], silicon nanowires [1,11,12], platinum and gold nanowires, and metal oxide nanowires [13]. However, fabrication of these nanowires is expensive and often requires harsh conditions [14]. Moreover, the inability to grow these nanowires in a site-specific location has limited their application.

Conducting polymer nanowires (CPNWs) have recently emerged as an attractive alternative to metal and semiconducting nanowires for their large conductivity change, flexibility, and ease of synthesis [15,16]. Furthermore, the CPNWs can be synthesized site-specifically at the desired location [16,17,18,19,20].

The discovery of the first organic polymer that possesses metallic conductivity, polyacetylene, in 1977 [21] has opened up possibilities for the development of a new class of materials known as intrinsically conducting polymers, or simply conducting polymers. In 2000, the Nobel Prize for Chemistry was awarded to the three scientists who discovered polyacetylene (Alan G. MacDiarmid, Hideki Shirakawa, and Alan J. Heeger) “for the discovery and development of conducting polymers”. Since then, a surge of research effort has been directed towards the development, synthesis, and characterization of new types of conducting polymer materials. As a result, many different kinds of conducting polymers have been discovered and synthesized. Some of the most well-known examples are polyaniline, polypyrrole, polythiophene, poly(3,4-ethylenedioxythiophene) (PEDOT), to name a few. In particular, polyaniline is one such organic polymer which has been known for over 150 years but was revisited in the 1980s and discovered that it possesses an electron conducting nature. Because of its tendency to be synthesized in a 1-dimensional nanofiber-like morphology, combining its environmental stability [22,23] and reversible redox chemistry, it has emerged as an excellent candidate to be used as a material for various nanoscale applications, particularly for chemical and biological sensors [24,25,26].

## 2. Polyaniline Nanowires: An Overview

Polyaniline is one of the oldest known conducting polymers [27] and has been extensively reviewed [28,29,30,31,32]. It has been the most studied conducting polymer closely followed by polypyrrole [33]. First discovered in the 19th century, polyaniline was originally known as “aniline black” [29,30]. It was later found to be electrically conductive in nature and many researchers began to closely examine the properties of this material. Polyaniline was initially grown as thin films but later it was discovered that, under certain conditions, it can be grown in the form of an interwoven nanowire network. It has been reported that polyaniline has an intrinsic nature to grow 1-dimensionally [34] which is not the case for other types of conducting polymers such as polypyrrole or polythiophene. Its unique ability to easily form 1-dimensional nanostructures, including wires, rods, tubes, and ribbons presents many advantages in nanoscale devices [4]. Polyaniline has been studied for use in a wide range of applications [35] such as chemical sensors, battery electrodes [36], supercapacitors [37], fuel cells [38], display devices [39], separation membranes [40] and anticorrosion coatings [41].

Protonation of the polymer, also known as doping, is the mechanism by which polyaniline becomes electrically conductive. However its conductivity is influenced by various factors including its electrochemical redox state, pH, humidity, temperature, and the type of anions in the solution [42]. The conductivity of polyaniline, in its pressed-pellet form, is typically in the range 2–10 S/cm [43].

To use polyaniline nanowires as chemiresistive sensors in the solution phase, a thorough knowledge of the polymer, especially its chemical backbone structures, conductivity and its dependence on the redox state, pH, and synthesis method among other points is needed. Therefore, in this section, a review covering the above mentioned topics is presented.

### 2.1. Chemical Structure and Electrochemical Properties

**Figure 1:**
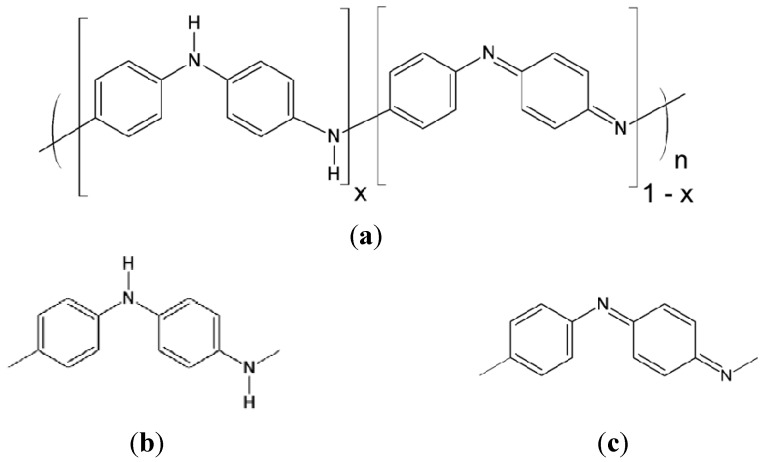
A general chemical structure of polyaniline and its repeating units. (**a**) A general chemical structure of polyaniline, (**b**) Reduced repeating unit, and (**c**) oxidized repeating unit.

The two most important factors determining the chemical structure of polyaniline are the redox state and the doping level. Polyaniline mainly has three distinguishable oxidation states, namely, the fully reduced (leucoemeraldine), the half oxidized (emeraldine), and the fully oxidized (pernigraniline) state, with virtually an infinite number of possible oxidation states existing in between. Therefore, in principle, polyaniline can exist in a continuum of oxidation states ranging from a completely reduced to a completely oxidized form. A general chemical structure of polyaniline is shown in Figure 1 where the polymer chain consists of two types of repeating units, namely, the reduced and the oxidized unit. The degree of oxidation is described by the variable *x* whose value is between 0 and 1, and it represents the fraction of the two repeating units.

**Figure 2:**
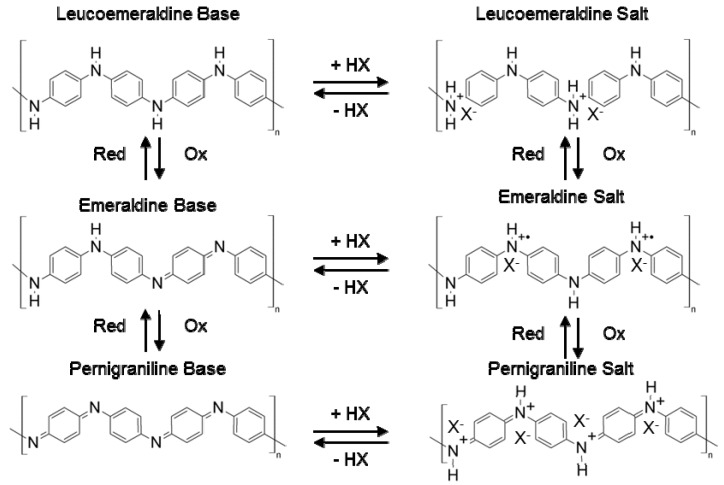
Electrochemical redox states and the corresponding doped form of polyaniline.

It follows therefore that leucoemeraldine, emeraldine, and pernigraniline refer to the chemical formula where *x* = 1, 0.5, and 0, respectively. The reduced repeating unit contains only the amine nitrogen atoms whereas the oxidized repeating unit is made up of imine nitrogen atoms only. The imine nitrogen atoms, which do not have hydrogen bonds, can be protonated in an acidic environment where protons can be attached to these nitrogen atoms to generate radical cations [44,45]. The degree of protonation strongly depends on the oxidation state of polyaniline and the pH of the aqueous solution in which the polymer is immersed. It was originally thought that protonation occurred exclusively on the imine nitrogen atoms, however, experimental results show that some amine nitrogen atoms can also be protonated to give NH_2_^+^ groups even if all the imines are not protonated [28,46,47,48]. An unprotonated polyaniline is known as a base whereas the protonated polymer is called a salt. The three different redox states of polyaniline in their base forms and the corresponding salts are illustrated in Figure 2.

**Figure 3:**
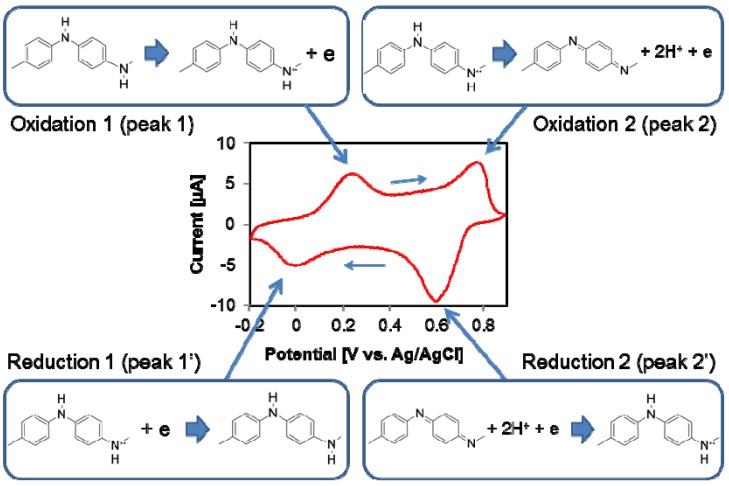
A typical cyclic voltammetry (CV) curve of polyaniline in HCl (pH 1) showing two sets of redox couples. The direction of potential scan is shown with the arrows.

The electrochemical behavior and the redox states of polyaniline are typically studied using cyclic voltammetry (CV). The CV response of polyaniline has been well documented [31,49,50] and some of the key results are described here. The electrochemical behavior of polyaniline is dependent on many parameters including the applied potential, the choice of material and the surface area of the electrodes, composition of the electrolyte, and temperature, among others. A typical CV curve of a synthesized polyaniline is shown in Figure 3.

The CV curve shows two sets of distinct redox activity as indicated by the two pairs of anodic and cathodic current peaks. The first set of a redox couple which appears between 0 and 0.25 V *vs.* silver/silver chloride reference electrode (Ag/AgCl) is associated with the conversion of the fully reduced leucoemeraldine base to the partially oxidized emeraldine, and the second set of redox current peaks occurring between 0.6 and 0.8 V *vs.* Ag/AgCl pertains to the conversion of emeraldine to the fully oxidized pernigraniline form. The potential of the first redox couple (peaks 1 and 1’) is largely independent of the pH whereas the potential for the second redox couple (peaks 2 and 2’) is strongly dependent on the pH value. This indicates that protons are involved in the redox reaction associated with the second peaks while the redox reaction related to the first anodic peak does not require protons as part of the reaction. Another point to note is that polyaniline is more easily oxidized in less acidic solutions, and this can be experimentally verified where the peaks 2 and 2’ shift to the left as the pH increases. Irreversible degradation also occurs at the second oxidation peak especially in strong acids. A linear relationship has been observed between peak height of the redox current and scan rate in a solution containing aniline and sulfuric acid solution which is indicative of an electron transfer limited process [51].

Using the CV data and the electrochemistry, the structural formula for polyaniline as it goes through the redox process can be interpreted. The following reactions have been proposed by Focke *et al.* [42] and a more detailed account can be found in [31]. Since the peak position of the first redox process in the CV plot is independent of pH, no proton is involved in the reaction. Hence, the reduction reaction can be described as the following:

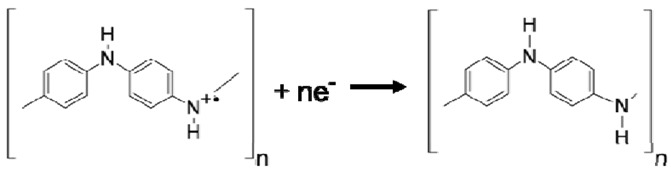



with *E*_o_ = 0.1 V *vs.* Ag/AgCl. However, the peak position of the second redox process is highly dependent upon the pH of the solution. The second oxidation peak and its corresponding reduction peak tend to move to more negative values at a rate of approximately 120 mV per pH unit as the pH is increased [31]. Since the peak position moves as a function of the proton concentration in the solution, it can be expected that protons are involved in the reaction as shown below:

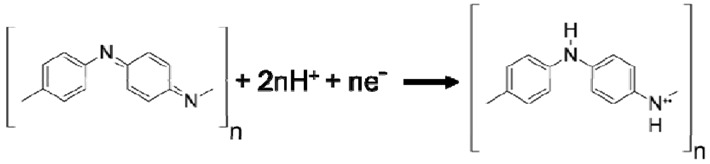



The doping mechanism of polyaniline is also unique among other conducting polymers. Most conducting polymers undergo redox doping processes during which the number of electrons associated with the polymer backbone changes. However, polyaniline can be doped through a non-redox process where the number of electrons in the polymer backbone structure remains unchanged [52], making the doping process simpler.

There are two ways that polyaniline can be doped, one is through acid treatment which is a non-redox doping process, and the other is by oxidation of leucoemeraldine to emeraldine which is a redox process. Upon proton doping, radical cations are formed at the imine nitrogen atoms and these charge carriers are believed to be responsible for the electronic conduction in polyaniline [53]. Hence, the majority charge carriers in polyaniline are holes.

### 2.2. Electronic Conduction

**Figure 4:**
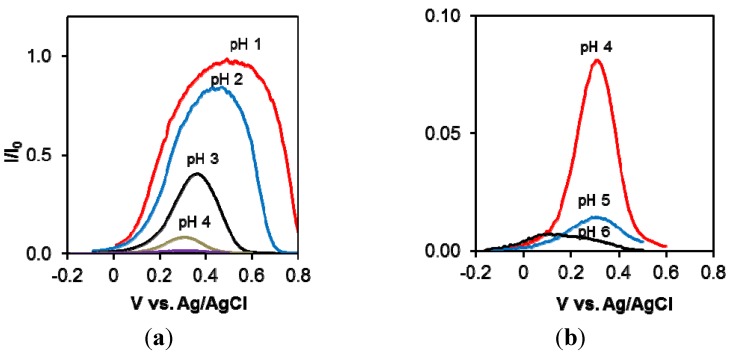
Conductance current *vs.* potential of polyaniline in various pH solutions: pH range 1–4 (**a**) and pH range 4–6 (**b**). I_0_ indicates maximum current observed.

Since the delocalized positively charged free radicals are the main source of conduction in polyaniline, it can be expected that maximum conductivity is exhibited when the number of radical cations in the polymer chains is maximized. This is in agreement with the fact that both leucoemeraldine and pernigraniline, neither of which have free radicals in their backbone structure, are completely insulating. Hence, the conduction current *vs.* the electrochemical potential relationship shows a bell shaped curve as illustrated in Figure 4. The graph also confirms that the most conductive form of polyaniline is the fully protonated, half-oxidized emeraldine salt form, and the conductivity decreases as the polymer is deprotonated or the oxidation state changes toward either a fully oxidized or a fully reduced state. Over-oxidation of polyaniline by applying potentials beyond +0.6 V *vs.* Ag/AgCl should be avoided because it causes the irreversible formation of quinonediimine structures which are electrochemically inactive [42,54,55].

**Figure 5:**
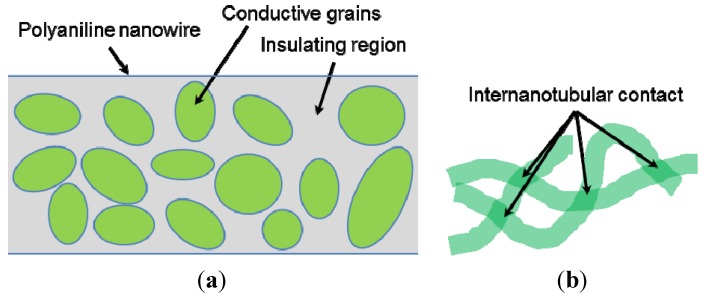
The electronic conduction path of the polyaniline nanowires: (**a**) internanotubular contacts between polyaniline nanowires, and (**b**) conductive granular region encapsulated in the insulating region of the nanowire. The drawings were adopted from [60].

Although a true mechanism of electron transport is still under debate, many theories have been proposed to explain the electronic conduction of polyaniline. It is generally accepted that polyaniline nanowires consist of pockets of conductive grains embedded in an insulating region as illustrated in Figure 5a. Since the conductive grains are separated by an insulating medium, electrons must gain sufficient energy to be able to overcome the insulating barrier and “hop” into the nearest neighboring conductive grain. Some models also take into consideration electronic conduction through internanotubular contact as illustrated in Figure 5b.

To further elucidate this phenomenon, many different models to describe the electron transport in polyaniline nanowires have been proposed, including granular-rod model [56], 3-dimensional variable range hopping (3D VRH) [57,58], 1-dimensional variable range hopping (1D VRH) with interchain coupling [59], Efros-Schklovskii (E-S) hopping conduction [60], and charging energy limited tunneling (CELT) [61]. A recent survey indicates that the true nature of the conduction mechanism must be examined on a mesoscopic scale [62].

## 3. Synthesis of Polyaniline Nanowires

There are many techniques available for synthesizing 1-dimensional polyaniline nanowires. The two most common techniques are chemical synthesis where polyaniline nanowires are formed by chemical oxidation of aniline monomers, and electrochemical polymerization where aniline monomers are electrochemically oxidized and polymerized on the surface of the anode of the electrochemical cell. Although other methods such as electrospinning [63], enzyme assisted growth [64], and DNA template method [65] exist, in this review, emphasis will be placed on the chemical and the electrochemical polymerization methods.

Various shapes and forms of polyaniline nanowires have been obtained, however the mechanism of the formation of such nanowires has not yet been fully elucidated [66,67,68]. Although the exact growth mechanism is uncertain, there have been many reports suggesting the probable growth mechanisms based on experimental results [29,49,69,70].

### 3.1. Chemical Synthesis

The classical chemical synthesis involves the direct oxidation of aniline monomers by chemical oxidants [29]. Polyaniline nanofibers can be obtained by mixing aniline monomers with a strong oxidizing agent in an acidic environment [71,72]. The most commonly used oxidant in chemical synthesis is ammonium peroxydisulfate (APS) [73]. However, using APS creates a harsh synthetic condition due to its strong oxidizing nature. Therefore, more environmentally benign synthesis methods that use a relatively mild oxidant, such as hydrogen peroxide with catalysts, instead of APS have been reported [64,73]. Depending on the growth condition, chemical synthesis can yield various shapes and forms of polyaniline nanostructures such as irregularly shaped agglomerates, granular particles, and elongated nanofibers. It has been experimentally observed that, during the early stage of polymerization, only 1-dimensional nanofibers were observed due to the homogeneous nucleation of polyaniline molecules. However, as further polymerization proceeds, preferential growth on previously formed nanowires by heterogeneous nucleation occurs, resulting in irregularly shaped particles. Therefore, suppression of this ‘secondary growth’ that takes place during the later stage of polymerization is likely to be the key to growing directional nanowires.

**Figure 6:**
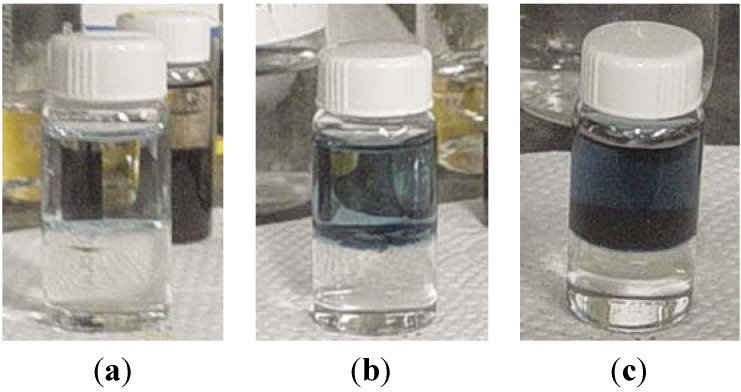
Interfacial polymerization process with 0.32 M of aniline in chloroform (bottom layer) interfacing 0.08 M of ammonium peroxydisulfate (top layer): (**a**) 1 min, (**b**) 5 min, and (**c**) 10 min of the reaction time after the reaction started.

Two novel synthesis methods that yield highly directional polyaniline nanowires with controllable diameters have been suggested: the first method is interfacial polymerization [74,75], and the second method is rapid mixing technique [72,76]. In the interfacial synthesis method, the polyaniline polymerization only occurs at the boundary of two immiscible organic/aqueous solutions. The synthesized polyaniline nanowires at the interface of the two solutions migrate into the bulk of the aqueous phase, thereby avoiding further polymerization. Figure 6 illustrates the early, the intermediate, and the final stages of the interfacial polymerization process. The downside of this technique is that the yield of nanofibers produced is generally too low to be mass produced on a large scale. On the basis of the experimental results that a fast reaction rate promotes homogeneous nucleation, and that conducting the reaction at a raised temperature (for example, 60 °C) produces less granular particles, a rapid mixing method was suggested. By quickly mixing aniline monomers with an appropriate portion of the oxidant at room temperature or higher, one can obtain large quantities of highly directional polyaniline nanofibers. The diameter of the nanowires is dependent on the type of acid used in the polymerization process. The average diameter of nanowires synthesized with HCl is approximately 30 nm while those obtained with camphorsulfonic acid (CSA) are roughly 50 nm, and HClO_4_ yielded an average diameter of 120 nm [74].

Since the nanofiber morphology of polyaniline can be intrinsically grown, templates are not necessary for 1-D polyaniline synthesis although there have been reports that use porous templates for the synthesis of nanowires [77,78]. There are other techniques available that yield the growth of polyaniline nanowires without the aid of templates including oligomer-assisted polymerization, seeding polymerization, falling pH polymerization, and dilute polymerization [79].

### 3.2. Electrochemical Synthesis

Polyaniline can be synthesized by anodic oxidation of aniline monomers through an inert electrode [29]. One of the key advantages of the electrochemical method is that this technique allows direct deposition of polyaniline nanostructures onto metal electrodes in a simple and cost effective way which ensures good ohmic contact between the electrode and the polymer. The nanowires are also addressable since the location of polymer growth is designated by the shape and pattern of the working electrode. Electropolymerization of polyaniline can be categorized into three types: potentiostatic, galvanostatic, and potentiodynamic growth.

#### 3.2.1. Potentiostatic Growth

**Figure 7:**
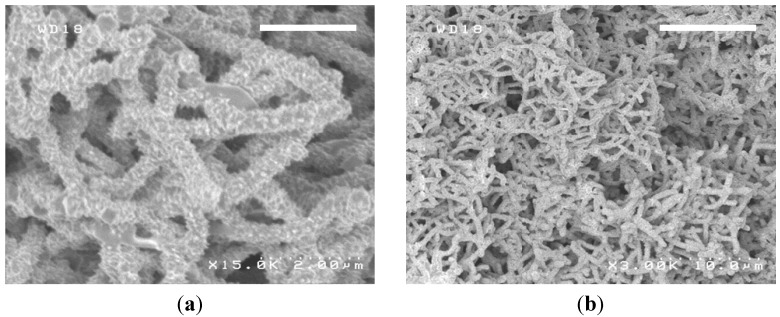
SEM images of the electrochemically synthesized polyaniline nanowires. Scale bars are 2 μm (**a**) and 10 μm (**b**).

In the potentiostatic method, polyaniline nanowires can be grown by applying a constant oxidative potential to the anode of the electrochemical cell, which causes polymerization of aniline on the surface of the anode. Polyaniline can be grown on a variety of metallic surfaces including platinum, gold, stainless steel, iron, copper, zinc, ITO, graphite, and glassy carbon among many others [30,35]. The electrolyte solution is generally a mixture of a strong acid (such as 1 M H_2_SO_4_, HCl, or HClO_4_) and aniline monomer with a concentration in the area of 0.05 M. The potential under which polyaniline nanowires can be grown is between 0.7 and 0.8 V *vs.* Ag/AgCl. The oxidation of the aniline monomers occurs at around 0.7 V. These oxidized species are thought to be in radical forms that quickly become *p*-aminodiphenylamine dimers. These formed dimers are much more easily oxidized than aniline monomers (around 0.2 V), and can be further oxidized to form longer chains of polyaniline. Therefore, once these dimers are formed, polymerization of aniline can proceed even at potentials lower than 0.7 *vs.* Ag/AgCl as long as the electrode surface is preliminarily covered with small amounts of polyaniline [80]. Typical polyaniline nanowires grown under the potentiostatic method are shown in Figure 7.

#### 3.2.2. Galvanostatic Growth

Galvanostatic growth is a method of electrochemical polymerization where the current flow in the working electrode is maintained at a constant value. A well-established low current polymerization technique involves a three step galvanostatic growth method [81]. In the first step, the current density is fixed to 0.08 mA/cm^2^ for 0.5 h which generates particles to act as nucleation sites for growing extended polymer nanostructures. This process is followed by the second step where the current density is reduced to 0.04 mA/cm^2^ for 3 h. During this stage, continued polymerization occurs at a slower growth rate. Finally in the third step, the current density is further reduced to 0.02 mA/cm^2^ for 3 h, which continues to elongate the nanostructures into wires. Typical diameters of these nanowires are 50 to 70 nm with approximately 0.8 μm in length. For producing longer nanowires, higher current density which promotes faster growth rate is required. Further, galvanostatically grown polyaniline is less stable due to the formation of quinoid structures which contribute to the final formation of the degradation product, *p*-benzoquinone, which prevents further growth of the polymer [82,83].

#### 3.2.3. Potentiodynamic Growth

Potentiodynamic growth involves scanning the potential of the working electrode linearly from initial to final value in a forward and reverse direction repeatedly until the desired amount of polymer has been deposited [84,85]. The sweeping voltage technique for the production of polyaniline is useful for elucidating basic aspects of the polymer growth and the redox mechanism [51]. It has been reported that continuously cycling the potential of the working electrode produces a more uniformly deposited polyaniline film compared to that formed at constant potential and promotes better adhesion to the electrode surface [86].

#### 3.2.4. Other Growth Methods

Other methods of polyaniline synthesis include the electrospinning technique [63,87,88] and the template-guided method. The hard template approach was proposed by Martin [77,78,89,90,91] and has been reviewed in detail [92]. DNAs have also been used as a template for the synthesis of polyaniline [65,93,94]. Horseradish peroxidases have been used as catalysts for the oxidation of aniline monomers in the presence of hydrogen peroxide to synthesize polyaniline [64,95]. Wang *et al.* have used an *in situ* electrochemical growth method to produce polyaniline and polypyrrole nanowires within a microfluidic device [16].

## 4. Polyaniline Nanowire-Based Chemiresistive Sensors

The applications for polyaniline nanowires are being actively explored by many researchers due to a number of useful properties such as direct and easy deposition on the electrode, control of thickness, high surface area, long term environmental stability, and tunable electrical conductivity [25]. Although our focus is on the development of a chemiresistive sensor that measures the change in conductivity of polyaniline caused by a reaction of interest, other detection mechanisms are also briefly discussed followed by a general survey of existing polyaniline-based chemiresistive sensors.

### 4.1. Potentiometric, Amperometric, and Conductometric Sensors

A potentiometric sensor measures the change in potential caused by a chemical reaction that separates electric charge. In general, the potential difference is in a logarithmic relationship with the activity of the target analyte as given by the Nernst equation. Examples of potentiometric sensors include ion-selective field effect transistors (ISFETs) [96,97,98], ion-selective electrodes (ISEs), and conventional pH meters. If there is a large difference between the activity and the concentration of the species of interest, then the potentiometric sensor may give inaccurate results. Amperometric sensors measure the current generated at the surface of the electrode as a result of electron transfer from the oxidation or reduction reaction of the electroactive species, typically at a constant applied potential. If the applied potential is not a constant but contains a change in potential, such as a step change, a pulsed voltage, or a linear sweep, then these types of sensing are referred to as voltammetric sensors. The current must vary linearly with respect to the concentration of the analyte in order to have accurate readings. Moreover, if two or more species in the sample solution have similar redox potentials then the generated electrochemical current may contain a substantial amount of interference which may compromise the selectivity of the sensor. Conductometric or resistive sensors measure the change in resistivity of the transducing material due to chemical reactions. Regardless of the type and the detection mechanism of the sensors, the important considerations inherent to all devices include, but are not limited to, sensitivity (limit of detection), selectivity (minimum false negative/positive signals), response time (fast reading), stability (short term and long term), and hysteresis (same measurement regardless of the past history of the sensor). Although polyaniline has been applied in all three types of sensors, to be able to truly make use of the tunable conductivity nature of polyaniline as a transducing mechanism, polyaniline must be used as a conductometric sensor. Chemiresistors and chemically sensitive field effect transistors (chemFETs) are the two main examples of conductometric sensors. In the following sections, polyaniline nanowire based sensors are reviewed. Table 1 summarizes the chemical [3,15,17,99,100,101,102,103,104,105,106,107,108,109,110] and biological [94,111,112] sensors that utilize polyaniline nanowires as sensing materials.

### 4.2. Gas Sensors

The importance of a reliable and accurate gas monitoring system is well understood not only from the safety but also from the environmental standpoint. Most commercially available gas sensors are based on metal oxide semiconducting materials (such as tin oxide) operating at high temperature to increase sensitivity. The use of conducting polymers as an alternative to inorganic semiconducting materials for the gas sensitive layer offers many advantages such as, low cost, ease of synthesis, tunable conductivity, fast response due to porosity of the material [113], and high sensitivity at room temperature. Polypyrrole was one of the first conducting polymers to be applied for gas sensing materials [114]. However, its low sensitivity, slow response, lack of adsorption-desorption reversibility has limited its use. Hence, the search for a more reliable and better performing material was extended to polyaniline. Huang *et al.* developed a chemically synthesized polyaniline nanofiber-based gas sensor and studied its response to 100 ppm of HCl and NH_3_ vapor [3,75,115]. Upon exposure to HCl vapor the resistance of the polyaniline nanofibers reduced through the doping process, and exposure to NH_3_ had a dedoping effect which increased the resistance of the polymer [99]. They also demonstrated that the nanofiber based sensor responded much faster than the conventional film to both doping and dedoping due to the highly porous morphology of the nanofiber film with the small diameter of the fibers resulting in faster diffusion of the gas molecules. Similar work was done in [17] where a polyaniline nanowire framework was formed to bridge the electrode junctions which acted as a resistive sensor, and its responses to HCl, NH_3_, and ethanol vapors were demonstrated. A polyaniline chemiresistors have also been developed for various gas sensors [15,100,101,116] as well as humidity sensors [102,103]. Combining polyaniline nanowires with other nanomaterials such as carbon nanotubes [104,117,118], graphenes [105], and gold nanoparticles [106] to form a composite layer has been shown to improve the sensitivity as well as the carrier mobility of the gas sensing material.

Separation of a gas species of interest from a mixture of gases is essential in improving the performance of a gas sensor in terms of sensitivity, selectivity, and protection of the sensing material from unwanted molecules. One of the most promising techniques to achieve the separation of gas is a membrane-based gas separation method. Separation of gas generally requires a membrane with pore sizes of the order of 3 nm or less [119]. Polyaniline-based membranes have been reported to be the most effective since their pore size can be controlled via the doping process either before or after the synthesis of the membrane. Polyaniline is an ideal choice for this application due to its simple doping/dedoping chemistry and its stability in air. The earliest work on polyaniline-based membranes for gas separation was reported in [40] where the porosity of the membrane was tailored by controlling the level of doping as well as the size of the dopant counterions. In a more recent work, composite membranes such as polyaniline/carbon nanotube [120], polyaniline-polysulfone [121], and polyaniline/polypropylene [122] have also been reported to have improved permeability and selectivity.

**Table 1:** Summary of chemical and biological sensors based on polyaniline nanowires and their derivatives.

Sensor Type	Sensing Material	Diameter (nm)	Analyte	Detection Limit (LOD)	Response Time	References
Surface acoustic wave	Polyaniline/In_2_O_3_	90	H_2_, NO_2_, CO	~ 2 ppm	~ 30 s	[107]
Surface acoustic wave	Polyaniline	30–50	H_2_	0.06%	~ 100 s	[108,109]
Amperometric	Polyaniline	60–80	nitrite	5 × 10^−8^ M	~ 5 s	[110]
Amperometric	Polyaniline/Au nanoparticles	30–50	glucose	5 × 10^−7^ M	~ 5 s	[111]
Chemiresistive	Polyaniline/CSA	~100	H_2_	<1%	-	[15]
Chemiresistive	Polyaniline	40–80	NH_3_, HCl, EtOH	~0.5 ppm	~100 s	[17]
Chemiresistive	Polyaniline/Au nanoparticles	80	H_2_S, CH_3_SH	~1 ppm	~20 s	[101]
Chemiresistive	Polyaniline	30–40	CO	~1 ppm	~100 s	[100]
Chemiresistive	Polyaniline	335	NH_3_	0.5 ppm	~75 s	[99]
Chemiresistive	Polyaniline	30–120	HCl, NH_3_, N_2_H_4_, CHCl_3_, CH_3_OH	100 ppm	2~200 s	[3]
Chemiresistive	SWCNT/Polyaniline	15	NO_2_, H_2_S	500 ppb	~10 min	[104]
Chemiresistive	Graphene/Polyaniline	25–50	H_2_	-	~1 min	[105]
Chemiresistive	Polyaniline/Au nanoparticles	250–320	H_2_S	0.1 ppb	<2 min	[106]
Chemiresistive	polyaniline	100	humidity	-	~1 min	[102]
Chemiresistive	Polyaniline/PVB/PEO	100	humidity	-	~50 s	[103]
Target-guided formation method	Polyaniline	-	microRNA	5 fM	30–60 min	[94]
Labeled direct charge transfer	polyaniline	~200	*Bacillus cereus*	~10 CFU/mL	-	[112]

### 4.3. Biosensors

The development of biosensors has emerged as a topic of great importance due to their applications in clinical diagnostics, environmental monitoring, food safety, and defense against biological warfare. An affinity based molecule recognition method which uses the specific interactions of biomolecules such as antibodies to antigen binding, DNA hybridization, enzyme catalysis, is the most common method to ensure high specificity and selectivity of a biosensor. One of the earliest biosensors was developed by Clark and Lyons [123] who used glucose oxidase immobilized on a semi-permeable membrane, and glucose was monitored by measuring the oxygen consumed by the enzyme catalytic reaction. The glucose sensor is one of the most commercially successful electrochemical biosensors existing today. Most glucose sensors employ an amperometric measuring technique [111,124], however, this technology has major limitations if the sensor is to be made smaller or if lower concentrations of analyte are to be measured. One of the strategies to overcome this limitation was the use of microelectrochemical transistors [125]. These devices make use of conductive polymers whose conductivity changes by several orders of magnitude upon oxidation or reduction [126,127]. This property of conducting polymers can be used to amplify signals transduced by electrochemical reactions. Therefore, polyaniline is an excellent candidate for biosensor applications in this regard [5,24,25]. This idea has been realized with a glucose sensor by attaching or embedding enzymes such as glucose oxidase to the polyaniline film [128,129,130]. 

A nanoscale glucose sensor was implemented by bridging a 20–60 nm electrode gap with polyaniline [131]. Pal *et al.* used polyaniline nanowires as direct charge transfer (DCT) electrical transducers for the detection of foodborne pathogen, *Bacillus cereus* [112]. In this method, polyaniline nanowires are attached to each of the antibodies and when these modified antibodies bind to antigen to form a sandwich complex via lateral flow immunoassay method, the nanowires form a bridge between two open electrodes to give resistive measurements [112,132,133,134]. Forzani *et al.* developed a hybrid amperometric and conductometric chemFET-based sensor that can detect neurotransmitter dopamine even in the presence of ascorbic acid, an interference, whose concentration can be much higher than the analyte itself [135]. Polyaniline chemiresistors have also been applied as biosensors for the detection of microRNAs [94,136,137], urea [138,139], *E. coli* [140], and H_2_O_2_ [141].

## 5. Current Limitations of Polyaniline-Based Sensors

Although polyaniline nanowires have made great progress as chemiresistive sensors, the current limitations and challenges facing polyaniline nanowires as chemical sensors must be addressed in order for the technology to make further advancement.

One of the main limitations of polyaniline is the loss of conductivity in neutral and high pH environment. Since polyaniline requires a large amount of protons attached to the polymer to be electrically conducting, it is a very poor conductor when the pH is greater than 5, which significantly limits its application. This is especially critical for biosensors where most enzymatic and cellular activities are pH sensitive, and function properly only in neutral pH environment, typically between pH 6 and 8. Therefore, preventing the loss of protons bound to the polyaniline structure in neutral pH solutions is the key to maintaining conductivity in such an environment. There have been attempts to achieve this by attaching negatively charged anions to the polymer which will attract positively charged protons. MacDiarmid and Epstein developed a “self-doped” polyaniline which contains negatively charged sufonate groups covalently bound to the aromatic rings of polyaniline [142,143,144]. Such self-doped polyaniline can also be synthesized by electro-copolymerization of aniline with its ring-substituted derivatives such as aminobenzenesulfonate [145,146]. Another method to achieve increased conductivity at neutral pH is the polymerization of aniline with large molecular weight organic acids. In this case, large molecular sized anions are trapped within the polyaniline matrix, thereby maintaining the polymer’s electronegativity in order to attract protons. Camphorsulfonic acid [147,148,149], dodecylbenzenesulfonic acid (DBSA) [150,151], and poly(2-acrylamido-2-methyl-1-propanesulphonic acid) (PAMPSA) [152,153,154] were reported to be the most effective organic acids used for this purpose. Furthermore, the use of such organic acids enhances solubility of the polymer and improves solution processability. Long chain polymeric anions such as polyacrylic acid (PAA) [155,156] and polystyrene sulfonate (PSS) [157,158] have also been incorporated during the polymerization. These aforementioned techniques have extended the polymer’s electroactivity at the neutral pH environment, however its conductivity has only slightly improved. Further improvements of its conductivity in the physiological pH range are required in order to implement reliable biosensors.

Exposing polyaniline to elevated pH solutions such as pH 5 or higher causes an irreversible conductivity degradation of the polymer. Some of the possible causes for this conductivity degradation are structural damage due to mechanical stretching or twisting of the polymer chain caused by the electrostatic charge of the dopant, loss of anions to counter balance the positive charge gained by proton adsorption [159], and the production of quinone-hydroquinone couples [53,81]. Therefore, minimizing or compensating for the conductivity degradation is crucial in developing a repeatable polyaniline-based sensor and this still remains to be solved.

The conductivity of polyaniline is known to possess hysteresis, which is illustrated in the current versus potential sweep characteristics plot [127,131,160,161] where the current response to the potential sweep in the positive direction is different from that to the reverse sweep of the potential. This existence of hysteresis is more closely related to the level of doping than the electrochemical potential or the pH of the polymer [47] and this apparent hysteresis or “memory effect” has been attributed to structural relaxations [162]. Hence, a sensor calibration step is required to eliminate hysteresis in measurements. A simple way to calibrate is to deprotonate the polymer in strong base solutions. However, this method is inconvenient and can also accelerate the conductivity degradation as previously discussed. Therefore, a convenient and reliable self-calibration method for the development of a hysteresis-free polyaniline-based sensor is required. The authors have recently reported a potential self-calibration mechanism of a polyaniline-based chemical sensor by way of electrochemical resetting of the polymer [163]. The doping level of the polyaniline nanowires can be initialized by changing the redox state of the polymer to the fully oxidized form, and by returning the electrochemical potential to the initial redox state, the hysteresis can be minimized for subsequent pH measurement. Further characterization and improvement of the concept of electrochemical self-calibration mechanisms for reproducible and low hysteresis pH sensing is currently being investigated.

One of the main drawbacks of the polyaniline-based chemiresistive sensor, especially in gas sensing, is its relatively slow response time. As indicated in Table 1, the response times of polyaniline-based sensors are of the order of tens of seconds. For practical use, the response time must be reduced preferably to less than several seconds. Polyaniline composited with other nanomaterials such as carbon nanotubes, graphenes, or gold nanoparticles seems promising in this regard and has been shown to improve the response time as discussed in the previous section. Another issue with a polyaniline-based chemical sensor is its longevity. Due to natural degradation and loss of conductivity over time, the practical application of such devices is constrained by limited shelf life. These concerns must be addressed in the near future before any realistic commercial development can be made.

## 6. Conclusions

In summary, we have reviewed the basic principles and properties of polyaniline nanowires and their use in various sensor applications. The conductivity of polyaniline can be changed by a few orders of magnitude by varying its electrochemical potential and the solution pH. This unique property can be utilized for various chemiresistive or conductometric sensors. Moreover, the large surface area of the polyaniline nanowires makes this an ideal material for applications in high sensitivity gas sensors and biosensors. Conducting polyaniline nanowires have advantages over other metal or semiconducting nanowires for their low cost, ease of synthesis, and for the ability to locally or site-specifically fabricate the nanowires. However, some disadvantages may include fragility and structural degradation under harsh environments. The polyaniline nanowire-based sensors have been reported to have improved sensitivity and response time due to their nanoscale morphology. Some of the immediate challenges regarding polyaniline nanowires include improving their conductivity in the physiological pH range, preventing or minimizing the conductivity degradation, and minimizing the hysteresis effect. Much work is currently underway to address these issues with some areas already showing signs of success, and the number of applications for polyaniline nanowires is expected to increase in the future.
